# Kriegsauswirkungen auf Unternehmen: Energieabhängigkeit und Preiserhöhungen

**DOI:** 10.1007/s10273-022-3267-4

**Published:** 2022-09-16

**Authors:** Jannis Bischof, Philipp Dörrenberg, Davud Rostam-Afschar, Thomas Simon, Johannes Voget

**Affiliations:** 1 LS für ABWL und Unternehmensrechnung, Universität Mannheim, Schloss Ostflügel, 68161 Mannheim, Deutschland; 2 LS für ABWL und Betriebswirtschaftliche Steuerlehre, Universität Mannheim, Schloss Ostflügel, 68161 Mannheim, Deutschland; 3 Sonderforschungsbereichs (SFB) German Business Panel, Universität Mannheim, L9, 7, 68161 Mannheim, Deutschland; 4 LS für ABWL und Rechnungswesen, Universität Mannheim, Schloss Ostflügel, 68161 Mannheim, Deutschland; 5 LS für ABWL, Taxation & Finance, Universität Mannheim, Schloss Ostflügel, 68161 Mannheim, Deutschland

**Keywords:** D22, E30, Q41

## Abstract

Der Beitrag präsentiert die Ergebnisse einer fortlaufenden Unternehmensbefragung im Rahmen des German Business Panels zu den betriebswirtschaftlichen Auswirkungen des Ukrainekriegs. Die derzeitigen Entwicklungen belasten den Ausblick deutscher Unternehmen und führen zu Zurückhaltung bei Investitionen und Neueinstellungen. Mehr als die Hälfte der befragten Unternehmen gibt an, dass sie Gas direkt in der Produktion einsetzen oder durch eine Gasrationierung ihre Lieferketten gestört würden. Preiserhöhungen, die sich in der aktuell hohen Inflationsrate widerspiegeln, sind eine direkte Antwort auf den Kostendruck, der insbesondere durch die Entwicklung der Energiepreise und Störungen der Lieferketten entsteht.

## Abstract (en)

The results of a continuous firm survey (conducted by the German Business Panel) are presented to shed light on the economic implications of the Russian invasion in Ukraine for German firms. The current developments impact the economic outlook and lead firms to invest and hire less. More than half of all surveyed firms report that gas rationing would affect their production either directly or indirectly through their supply chain. Furthermore, one-half of firms report that it would require less than one year to adjust to a hypothetical situation in which it has 50 % less gas. However, more than a quarter of firms indicate an adjustment period that is longer than two years. Price increases are a direct response to cost pressure that is caused by the development of energy prices and supply-chain disruptions.

Im Rahmen des German Business Panels (GBP) werden Unternehmen in Deutschland fortlaufend zu verschiedenen Themengebieten befragt. Das GBP ist an der Universität Mannheim angesiedelt und Teil des durch die DFG geförderten überregionalen SFB „TRR 266 Accounting for Transparency“. Neben Befragungen, die sich schwerpunktmäßig mit Themen im Bereich Rechnungswesen und Besteuerung befassen, werden auf einer rollierenden Basis Befragungen zu aktuellen wirtschaftspolitischen Entwicklungen durchgeführt. Der Beitrag präsentiert die Ergebnisse einer Reihe von Umfragen, die zwischen Januar und Mai 2022 durchgeführt wurden und sich schwerpunktmäßig mit den Folgen des russischen Angriffskriegs für deutsche Unternehmen befassen. Die Ergebnisse basieren auf den Umfrageantworten von insgesamt etwa 2.800 Unternehmen, die in ganz Deutschland tätig sind und eine Vielzahl von verschiedenen Branchen abbilden. Die Befragungen werden online durchgeführt.[1]

1 Für methodische Details sowie für weitergehende Informationen etwa zur geografischen Abdeckung siehe https://gbpanel.org/ sowie Bischof et al. (2021).

* Wir danken dem Team des GBP. Ohne die exzellente Arbeit aller Teammitglieder wären die Implementierung, Durchführung und Betreuung der Firmenumfrage nicht möglich: Dirk Simons (PI), Laura Arnemann, Fabian Eble, Johannes Gaul, Christopher Karlsson, Yuhan Liu, Katharina Schmidt, Samhitha Srinivas, Tobias Kreuz und Paul Kimmann. Wir danken außerdem der DFG für die Förderung im Rahmen des überregionalen Sonderforschungsbereichs: Collaborative Research Center (SFB/TRR) Projektnummer 403041268 – „TRR 266 Accounting for Transparency“.

Einen besonderen Schwerpunkt legen wir auf die zentralen Fragen, wie genau die Unternehmen von einer möglichen Gasrationierung betroffen wären (was von Problemen beim Heizen der Gebäude bis zum direkten Einsatz von Gas in der eigenen Produktion reichen kann) und wie schnell die Unternehmen eine Gasrationierung kompensieren könnten (die Frage nach Substitutionsmöglichkeiten). Die Ergebnisse zu den Auswirkungen der Krise auf das Preissetzungsverhalten sind gerade im Kontext der Entwicklung der gesamtwirtschaftlichen Inflation bedeutsam.

## Entwicklung der Kennzahlen und Erwartungen

Die betriebswirtschaftlichen Erwartungen von Unternehmen in Deutschland sind unmittelbar nach dem russischen Angriff auf die Ukraine eingebrochen. Abbildung 1 verdeutlicht den starken Rückgang der unternehmerischen Umsätze, Gewinne und Investitionen seit Kriegsbeginn. Während die erwartete Gewinnwachstumsrate kurz vor Kriegsausbruch durchschnittlich noch bei knapp 4 % lag, ist diese bis Mitte April auf -6,6 % gefallen. Umsatzerwartungen (von 10,7 % auf 3,1 %) und Investitionsplanungen (von 7,7 % auf -2,1 %) sind in ähnlichem Maße zurückgegangen und konnten auch durch die Beendigung der bundesweiten Coronamaßnahmen Ende März nicht aufgehalten werden. Seit Mai 2022 zeigen die Daten des GBP nun eine Stabilisierung der betriebswirtschaftlichen Kennzahlen. Zuletzt rechnen die Unternehmen mit einem Wachstum der Umsätze und Investitionen von 8 % bzw. 2 % und einem Rückgang der Gewinne um nur noch -2,8 % gegenüber den Vorjahreswerten.

Der starke Einbruch der betriebswirtschaftlichen Kennzahlen infolge des Kriegsausbruchs ist mit den erwarteten weitreichenden Folgen des Kriegs und den bereits

Abbildung 1
**Entwicklung betriebswirtschaftlicher Kennzahlen**
in %

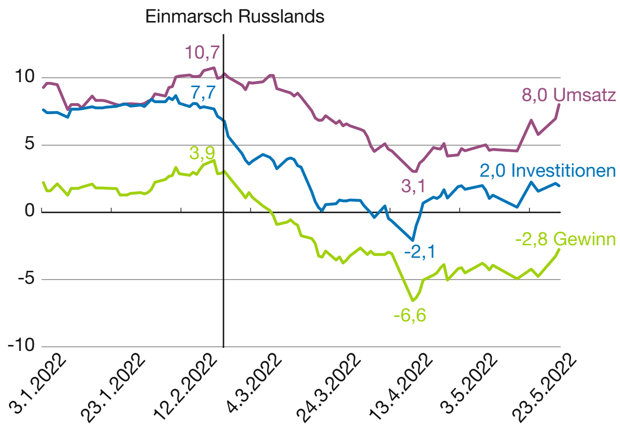

Quelle: eigene Berechnung auf Basis der Daten des GBP.

Abbildung 2
**Erwartete Belastungen durch den Krieg**
in %

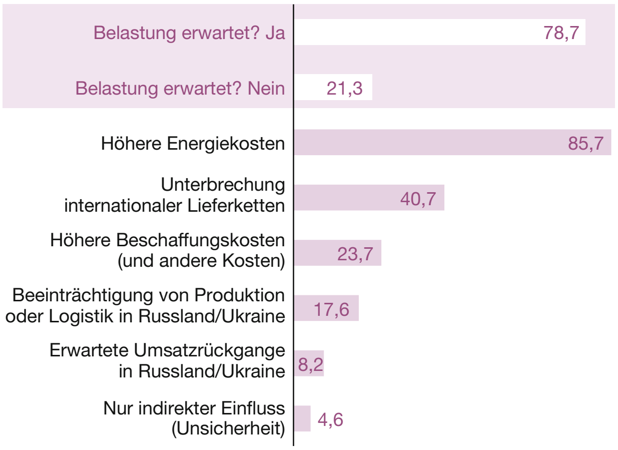

Quelle: eigene Berechnung auf Basis der Daten des GBP.

etablierten Sanktionen gegen Russland zu erklären: Abbildung 2 zeigt, dass lediglich 21 % der befragten Unternehmen keine finanziellen Belastungen durch den Krieg befürchten. Die vom Krieg betroffenen Unternehmen sorgen sich vor allem um steigende Energiekosten (86 %), Störungen internationaler Lieferketten (41 %) sowie höhere Beschaffungs- und Materialpreise (24 %). Beeinträchtigungen der Geschäfte in der Ukraine und in Russland (18 %) sowie dortige Umsatzeinbußen (8 %) betreffen hingegen eine deutlich geringere Zahl an Unternehmen.

Trotz dieser Belastungen stehen deutsche Unternehmen den bisherigen Sanktionen der deutschen Bundesregierung gegen Russland überwiegend positiv gegenüber: Zu Kriegsbeginn bewerten 32 % der Unternehmen die Sanktionen als positiv und 30 % sogar als sehr positiv. Lediglich 15 % der Befragten beurteilen die bestehenden Sanktionen negativ. Ein Importstopp von Rohstoffen wie Erdöl und -gas aus Russland trifft hingegen auf ein geteiltes Echo.

Der Anteil negativer Bewertungen eines Rohstoffembargos weist im Zeitverlauf einen eher steigenden Trend auf. Abbildung 3 stellt die Beurteilung eines Rohstoffembargos im Zeitverlauf dar. Während zu Kriegsbeginn noch mehr als 40 % der Unternehmen Unterstützung für ein Rohstoffembargo bekundeten, ist der Anteil positiver Bewertungen bis Anfang Juni auf 30 % gefallen. Am aktuellen Rand sprechen sich mehr Unternehmen gegen ein Rohstoffembargo aus (41 %). Diese Zahlen spiegeln indirekt auch wider, wie stark die Auswirkungen eines durch die russische Seite initiierten Lieferstopps die Unternehmen treffen würde.

Indes zeigt sich, dass es zwischen den einzelnen Wirtschaftszweigen große Unterschiede in der Bewertung eines möglichen Rohstoffembargos gibt. Abbildung 4 verdeutlicht dies für die Nutzung von Erdgas: Industrien, die sich stärker auf den Einsatz dieses Rohstoffs stützen (z. B. Bau und Verarbeitendes Gewerbe), bewerten einen Importstopp von Rohstoffen überdurchschnittlich negativ.

**Prof. Dr. Jannis Bischof** lehrt an der Universität Mannheim und ist Projektleiter im DFG-geförderten TRR 266 „Accounting for Transparency“ sowie Research Fellow am Leibniz-Institut für Finanzmarktforschung SAFE.

**Prof. Dr. Philipp Dörrenberg** lehrt Allgemeine Betriebswirtschaftslehre und Betriebswirtschaftliche Steuerlehre an der Universität Mannheim.

**Dr. Davud Rostam-Afschar** ist akademischer Rat an den Universitäten Mannheim und Hohenheim sowie akademischer Leiter des German Business Panels im DFG-geförderten TRR 266 „Accounting for Transparency“.

**Thomas Simon** ist wissenschaftlicher Mitarbeiter an der Universität Mannheim und im DFG-geförderten TRR 266 „Accounting for Transparency“ tätig.

**Prof. Dr. Johannes Voget** ist Inhaber des Lehrstuhls für ABWL, Taxation & Finance an der Universität Mannheim.

Abbildung 3

**Bewertung eines Gasembargos im Zeitverlauf**

in %

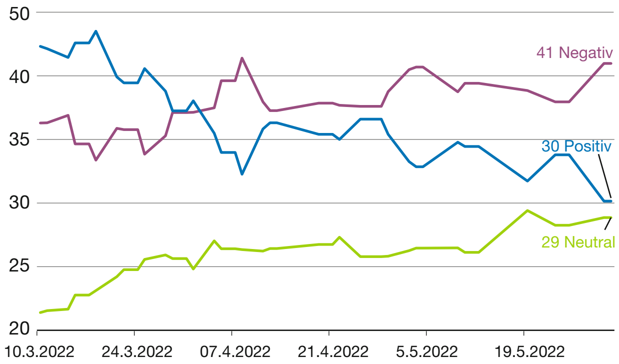

Quelle: eigene Berechnung auf Basis der Daten des GBP.

Abbildung 4

**Bewertung eines Rohstoffembargos**

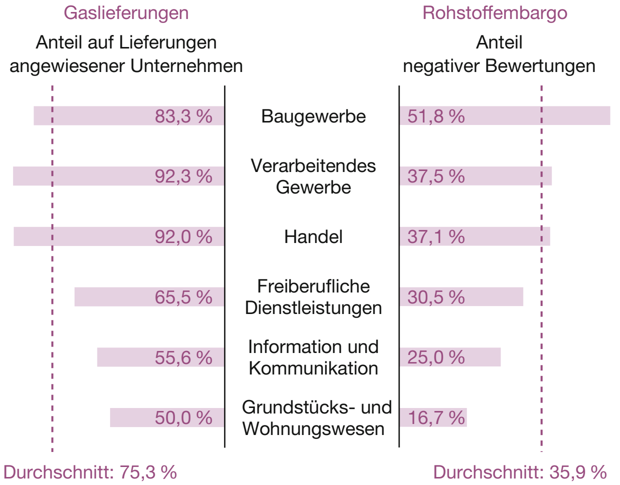

Quelle: eigene Berechnung auf Basis der Daten des GBP.

So liegt etwa der Anteil negativer Bewertungen eines Rohstoffembargos im Baugewerbe, das zu 83 % direkt oder indirekt auf Gaslieferungen angewiesen ist, bei mehr als 50 %. Deutlich positiver stehen einem Embargo hingegen Wirtschaftszweige gegenüber, die seltener auf die Nutzung von Gas angewiesen sind (und wenn, dann regelmäßig nur für den eigenen Heiz-, nicht aber den Produktionsbedarf), darunter der IT- und Dienstleistungssektor. In diesen Branchen beschränkt sich der Anteil negativer Bewertungen auf unterdurchschnittliche 25 % bzw. 31 %.

Wie eine Rationierung von verfügbarem Gas bei einem Ausfall der Lieferungen aus Russland die Unternehmen konkret betreffen würde, ist in Tabelle 1 aufgeschlüsselt. Zunächst zeigt sich, dass drei von vier deutschen Unternehmen durch eine Rationierung von Erdgas belastet würden: Der Anteil der auf Gaslieferungen angewiesenen Unternehmen im GBP liegt bei 75,3 % und somit höher als der Anteil von 50 % der Betriebsstätten, der in Kagerl et al. (2022) berichtet wird. Diese Unterschiede lassen sich aber durch methodische Unterschiede der Befragungen erklären (die Beobachtungseinheit im GBP ist das rechtlich selbstständige Unternehmen, im IAB-Betriebspanel hingegen die Betriebsstätte; das GBP nutzt zudem eine weitere gefasste Definition der Gasabhängigkeit unter Einbezug der gesamten Wertschöpfung). Die Art der Nutzung von Erdgas in den Geschäftsprozessen variiert stark. Während Erdgas nur von einem kleinen Teil der Unternehmen (3,4 %) direkt in der Produktion eingesetzt wird, geben 53 % der Unternehmen an, dass sie Vorleistungen von Unternehmen beziehen, die direkt von der Gasversorgung abhängig sind, und daher im Fall einer Gasrationierung Probleme entlang der Wertschöpfungskette befürchten. Die Hälfte der befragten Unternehmen nutzt Gas für Heizzwecke. 20,8 % aller Unternehmen nutzen Gas sogar ausschließlich für den eigenen Heizbedarf.

Unternehmen sind also auf sehr unterschiedliche Weise auf Gaslieferungen angewiesen. Tabelle 1 verdeutlicht die erwarteten Auswirkungen einer Gasrationierung – unter der Annahme, dass es infolge eines Importstopps zu einer Drosselung der Gaslieferungen um 50 % kommen würde. Der Gewinneinbruch fällt moderat aus (-4,9 %), wenn Gas für den Heizbedarf genutzt wird. Einen deutlich höheren Gewinneinbruch erwarten Unternehmen allerdings, wenn sie Gas in der Wertschöpfungskette als Vorleistung beziehen (-19,3 %) oder den Rohstoff selbst in der eigenen Produktion einsetzen (-48,3 %). Insbesondere hängt die Anpassungsfähigkeit der Unternehmen an eine Gasratio-

Tabelle 1

**Abhängigkeit von Erdgas**

in %

| Sind Sie auf Gaslieferungen angewiesen? | Anteil | Mittlerer Gewinneinbruch bei Rationierung | Anteil Anpassung an Rationierung länger als 2 Jahre | Anteil negativer Bewertungen eines Rohstoffembargos |
|---|---|---|---|---|
| Wir nutzen Gas direkt in der eigenen Produktion. | 3,4 | -48,3 | 33,3 | 83,3 |
| Wir nutzen Gas für unsere Heizung. | 50,6 | -4,9 | 7,4 | 43,8 |
| Wir erwarten Ausfälle unserer Lieferanten, da diese Gas nutzen. | 53,4 | -19,3 | 30,8 | 61,7 |
| Wir sind nicht auf Gaslieferungen angewiesen. | 24,7 | – | – | 22,7 |

Quelle: eigene Berechnung auf Basis der Daten des GBP.

Abbildung 5
**Anpassungsgeschwindigkeit in Abhängigkeit von der Erdgasnutzung**

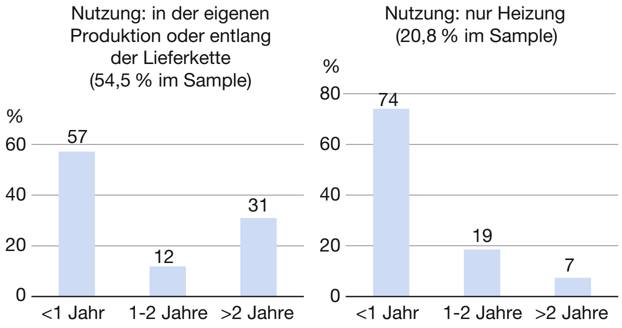

Quelle: eigene Berechnung auf Basis der Daten des GBP.

Abbildung 6
**Planung von Investitionen und Neueinstellungen seit Kriegsausbruch**

in %

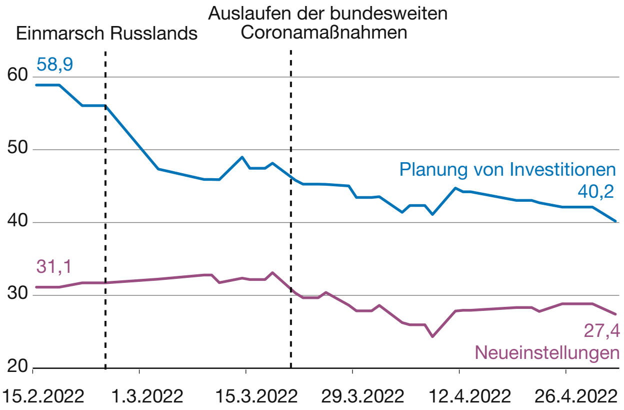

Quelle: eigene Berechnung auf Basis der Daten des GBP.

nierung von der Art der Gasnutzung ab. Wird Gas für den eigenen Heizbedarf eingesetzt, erwarten nur 7,4 % der Unternehmen, dass die Anpassung an die reduzierten Gasmengen mehr als zwei Jahre in Anspruch nimmt. Wenn Gas ein Produktionsfaktor in der Wertschöpfung ist, rechnen jedoch über 30 % der Unternehmen mit einer notwendigen Anpassung von mehr als zwei Jahren. Abbildung 5 stellt den erwarteten Anpassungszeitraum für beide Gruppen gegenüber. Die Nutzung für den Heizbedarf kann vergleichsweise schnell (<1 Jahr) ersetzt werden (für 74 % der Unternehmen). Jedoch erwarten nur 57 % der Unternehmen eine solch kurzfristige Anpassung, wenn sie auf Gas in ihrer Wertschöpfungskette angewiesen sind. Insgesamt gibt die Hälfte der Unternehmen an, dass sie für eine Anpassung an eine Situation mit 50 % geringerer Gasmenge weniger als ein Jahr benötigen würden. Ein Viertel rechnet jedoch mit einer Anpassungszeit von mehr als zwei Jahren.

## Auswirkungen auf unternehmerische Entscheidungen

Was bedeutet die Entwicklung der betriebswirtschaftlichen Kennzahlen und die finanziellen Belastungen infolge des Kriegsausbruchs für die konkreten Entscheidungen, die Unternehmen treffen? Abbildung 6 zeigt den Anteil an Unternehmen, die im Vergleich zum Jahr 2021 zusätzliche Investitionen planen und die Zahl ihrer Beschäftigten erhöhen möchten. Bei den geplanten Investitionen zeigt sich ein deutlicher Rückgang seit Kriegsbeginn: Wollten Mitte Februar noch beinahe 60 % der Unternehmen in Deutschland Neuinvestitionen tätigen, gilt dies zuletzt nur noch für etwa 40 %. Auch das Auslaufen der bundesweiten Coronamaßnahmen Ende März konnte diese negative Entwicklung nicht aufhalten. Die Zahl der Unternehmen, die zusätzliche Beschäftigte einstellen wollen, sinkt ebenfalls – wenngleich in etwas abgeschwächter Form: Während vor dem Krieg noch 31,1 % der Unternehmen Neueinstellungen vornehmen wollten, waren es zuletzt nur noch 27,4 %. Dies entspricht einem Rückgang um 12 %.

Wie sich die betriebswirtschaftliche Planung auf Branchenebene verändert hat, ist in Abbildung 7 dargestellt. Im Verarbeitenden Gewerbe fällt der Anteil an Unternehmen, die Neuinvestitionen planen, relativ moderat um 9 Prozentpunkte. Dagegen ist der Rückgang im Handel deutlich ausgeprägter: Hier geben seit Ende Februar nur noch 38 % der Unternehmen an, ihre Investitionen ausbauen zu wollen (gegenüber 55 % zu Jahresbeginn). Auch im Dienstleistungssektor und im Bereich der Information und Kommunikation geht der Anteil an Unternehmen mit wachsenden Investitionen deutlich zurück (-15 bzw. -13 Prozentpunkte). Die Unternehmen des Baugewerbes reagieren ebenfalls mit deutlichen Anpassungen ihrer betrieblichen Planung. Hier liegt der Anteil an Unternehmen, die Investitionen steigern und neue Beschäftigte einstellen, infolge des Kriegs nur noch bei 47 % bzw. 27 % und ist im Vergleich zu Jahresbeginn damit innerhalb von zwei Monaten um insgesamt 10 bzw. 13 Prozentpunkte gesunken.

Die Anpassungseffekte infolge des Kriegs fallen in den einzelnen Wirtschaftszweigen also unterschiedlich aus. Aber welche Faktoren beeinflussen, ob Unternehmen ihre betriebswirtschaftliche Planung in Reaktion auf den Kriegsausbruch verändern? Tabelle 2 stellt Unterschiede in den Entscheidungen von Unternehmen in Abhängigkeit davon dar, ob sie durch die bereits gegen Russland eingesetzten Sanktionen finanziell belastet werden, ob und inwiefern sie auf russische Rohstoffe angewiesen sind und ob sie eine Rationierung von Gaslieferungen schnell kompensieren könnten. Zunächst zeigt sich, dass Unternehmen, die finanzielle Belastungen durch die aktuell implementierten Sanktionen gegen Russland erfahren, im Mittel seltener investieren und einstellen wollen als Betriebe ohne finanzielle Belastungen durch den Krieg. Während durch

Abbildung 7
**Betriebswirtschaftliche Entscheidungen auf Branchenebene**

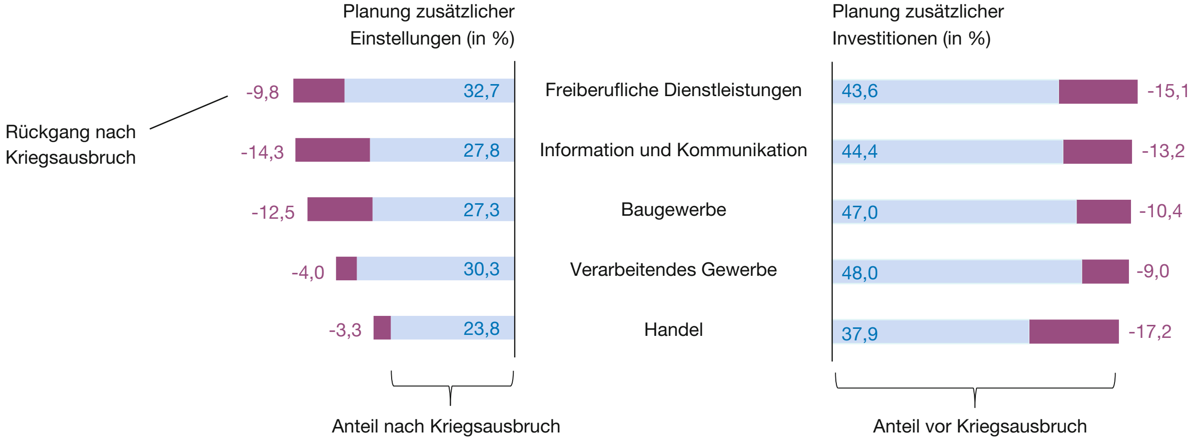

Quelle: eigene Berechnung auf Basis der Daten des GBP.

Sanktionen beeinträchtigte Unternehmen zusätzlich eine stärkere Neigung zu Preiserhöhungen und Investitionen in Forschung und Entwicklung zeigen, sind Ausschüttungen und Boni kaum durch aktuelle wirtschaftliche Beeinträchtigungen infolge des Ukrainekriegs beeinflusst.

Mehr als durch die bereits eingeführten Sanktionen gegen Russland und ihre wirtschaftlichen Folgen scheinen die unternehmerischen Entscheidungen allerdings durch die Unsicherheit über die künftige Energieversorgung beeinflusst zu werden. Differenziert man etwa danach, wie stark Unternehmen auf Erdgas in ihren Betriebsprozessen angewiesen sind, ergeben sich in Bezug auf Investitionen, Preis- und Liquiditätsentscheidungen eklatante Unterschiede. Tabelle 2 zeigt, dass die Bereitschaft für zusätzliche Investitionen bei Unternehmen, die Gas direkt im Produktionsprozess oder entlang der Wertschöpfungskette einsetzen, um 21 % geringer ist als bei Betrieben, die lediglich mit Gas heizen. Starke Abweichungen ergeben sich auch in Bezug auf andere zentrale betriebswirtschaftliche Entscheidungen: Unternehmen mit direktem Einsatz von Gas im Produktionsprozess wollen um 30 % eher Preise erhöhen und um 75 % eher Ausschüttungen kürzen.

Diese Unterschiede in der betrieblichen Planung hängen insofern auch damit zusammen, wie stark die Belastungen sind, die Unternehmen infolge eines Ausfalls der russischen Gaslieferungen erwarten – wodurch sie auch besonders stark der Inflation ausgesetzt sind – und wie flexibel sie ihre betrieblichen Prozesse an eine Gasrationierung anpassen können. Tabelle 2 zeigt, dass Unternehmen, die hohe Belastungen durch Kürzungen von Gaslieferungen erwarten und die Energieversorgung nicht innerhalb eines Jahres umstellen könnten, besonders stark auf die Krise reagieren, indem sie seltener investieren und einstellen, seltener ausschütten und häufiger Preise erhöhen. Diese Ergebnisse deuten darauf hin, dass Unternehmen mit einer hohen Abhängigkeit von Rohstoffen aus Russland bereits im Frühjahr mit einer Anpassung ihrer betrieblichen Planung reagiert haben.

### Ursachen für Preiserhöhungen

Obwohl sich die unternehmerischen Umsätze, Gewinne sowie Investitionen im Mai 2022 stabilisierten, wird die betriebswirtschaftliche Situation durch den anhaltenden Kostendruck belastet. Diesen Kostendruck geben Unternehmen zunehmend an ihre Kundschaft weiter. Abbildung 8 verdeutlicht, dass der Anteil an Unternehmen, die innerhalb der folgenden zwölf Monate Preiserhöhungen planen, zuletzt ein Rekordhoch erreicht hat. Nachdem sich das Preisniveau schon seit Jahresbeginn deutlich erhöht hat, gaben im Mai 2022 76 % der Unternehmen an, ihre Preise weiter erhöhen zu wollen (gegenüber 60 % im August 2021). Nur 4,3 % der Unternehmen planen, ihre Preise zu senken. Eine kurzfristige Entspannung der Preislage scheint somit nicht in Sicht.

Wie hoch die Neigung zu Preiserhöhungen in einzelnen Wirtschaftszweigen ist, zeigt Abbildung 9. Im Zeitverlauf wird zunächst deutlich, dass der Anteil preiserhöhender Unternehmen im Dienstleistungssektor (zuletzt 62 %) und im Bereich der Kommunikation und Information (zuletzt 55 %) durchgängig unter dem Gesamtdurchschnitt liegt. Im Baugewerbe, im Verarbeitenden Gewerbe sowie im Handel ist der Anteil an Unternehmen, die ihre Preise erhöhen, überdurchschnittlich hoch. Dabei ist im Baugewerbe zuletzt eine rückläufige Tendenz in Bezug auf Preiserhöhungen zu beobachten. Aufgrund steigender Zinsen können höhere Finanzierungskosten hier im zweiten Quartal 2022 zu einer geringeren Nachfrage führen und die Preise drücken. Eine solche abflachen-

Tabelle 2
### Einflussfaktoren der Entscheidungsplanung nach Kriegsausbruch in %

| | Zusätzliche Investitionen | Zusätzliche Beschäftige | Kürzung Ausschüttungen | Kürzung Boni | Kürzung F&E | Preiserhöhung |
|---|---|---|---|---|---|---|
| Gesamtsample | 45,1 | 29,9 | 31,1 | 27,3 | 10,5 | 72,9 |
| Belastung durch bisherige Sanktionen? | | | | | | |
| Ja | 41,4 | 26,2 | 30,8 | 27,0 | 13,6 | 76,8 |
| Nein | 44,7 | 33,5 | 31,9 | 27,8 | 6,1 | 65,3 |
| Betriebliche Bedeutung von Erdgas | | | | | | |
| Keine Abhängigkeit | 39,6 | 29,7 | 24,3 | 23,3 | 6,8 | 61,4 |
| in Produktion oder Lieferkette | 36,7 | 23,7 | 45,2 | 37,3 | 21,3 | 80,6 |
| nur für Heizung | 46,5 | 26,8 | 25,8 | 24,8 | 1,2 | 62,1 |
| Erwartete Belastung durch Embargo | | | | | | |
| Hoch | 38,7 | 22,2 | 46,3 | 41,3 | 22,4 | 82,7 |
| Niedrig | 40,8 | 29,1 | 32,0 | 23,1 | 5,6 | 68,7 |
| Substituierbarkeit von Gas: | | | | | | |
| Langsam (>1 Jahr) | 36,1 | 20,2 | 49,0 | 40,0 | 28,1 | 78,5 |
| Schnell (≤1 Jahr) | 43,6 | 29,9 | 39,1 | 31,3 | 9,9 | 75,0 |

Quelle: eigene Berechnung auf Basis der Daten des GBP.

de Entwicklung ist im Handel und Verarbeitenden Gewerbe nicht zu verzeichnen. In diesen Wirtschaftszweigen geben zuletzt jeweils mehr als 85 % der Unternehmen an, ihre Preise in den kommenden zwölf Monaten anzuheben.

Welche Faktoren beeinflussen die Preisentscheidungen von Unternehmen konkret? Abbildung 10 stellt dar, wie die Planung von Preiserhöhungen mit finanziellen Belastungen, die Unternehmen im Zuge des Ukrainekriegs erfahren, zusammenhängt. Die Analyse verdeutlicht, dass vor allem steigende Energiekosten einen bedeutenden Preistreiber darstellen. Während nur 48,5 % der Unternehmen, die nicht oder nur indirekt durch die Folgen des Kriegs beeinflusst sind, planen, Preise anzuheben, gilt dies bei Unternehmen mit steigenden Energiekosten für 73 %. Auch Störungen der Lieferketten erhöhen die Wahrscheinlichkeit für Preiserhöhungen um etwa 10 Prozentpunkte. Wenn Unternehmen durch Beeinträchtigungen der Geschäfte in Russland und der Ukraine belastet werden, steigt die Neigung zu Preiserhöhungen um 8 Prozentpunkte. Diese Angaben beziehen sich lediglich auf den Einfluss einzelner Belastungen. Ist ein Unternehmen von mehreren solcher Belastungen betroffen, erhöht sich die Wahrscheinlichkeit für Preiserhöhungen noch weiter.

Abbildung 8
### Geplante Preiserhöhungen

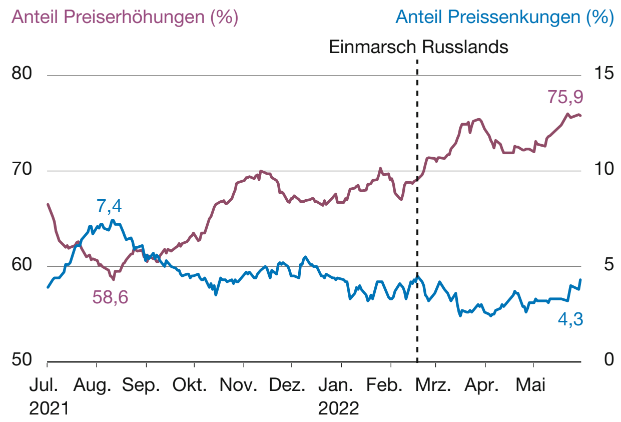

Quelle: eigene Berechnung auf Basis der Daten des GBP.

Abbildung 9
### Preiserhöhungen nach Wirtschaftszweigen in %

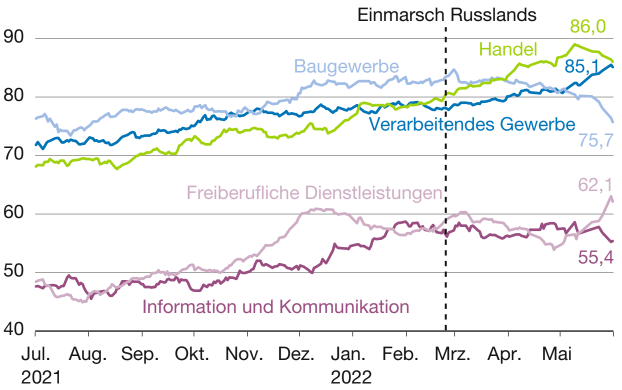

Quelle: eigene Berechnung auf Basis der Daten des GBP.

Abbildung 10
**Ursachen für Preiserhöhungen**

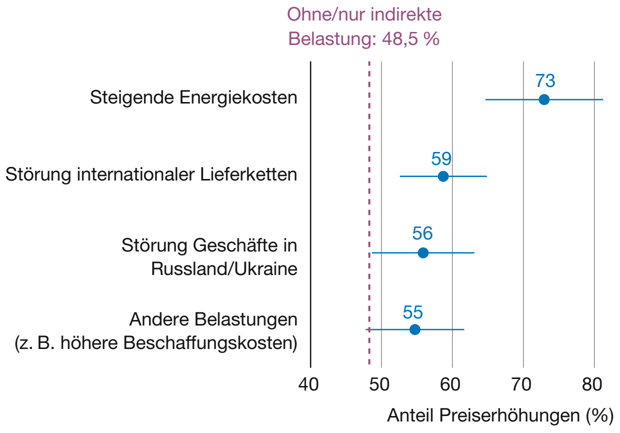

Quelle: eigene Berechnung auf Basis der Daten des GBP.

## Fazit

Der Ukrainekrieg und die damit verbundenen Entwicklungen auf den Energiemärkten und bei den weltweiten Lieferketten belasten den Ausblick deutscher Unternehmen und führen zu unternehmerischer Zurückhaltung bei Investitionen und Neueinstellungen. Eine Gasrationierung würde Unternehmen auf unterschiedliche Art und Weise treffen. Mehr als die Hälfte der befragten Unternehmen gibt an, dass sie Gas direkt in der Produktion einsetzen oder durch eine Gasrationierung ihre Lieferketten gestört werden. Diese Unternehmen erwarten starke Gewinneinbrüche im hypothetischen Fall einer eingeschränkten Gasversorgung. Auch sind diese Unternehmen weniger gut in der Lage, einen Gasausfall zu kompensieren und haben schlechtere Substitutionsmöglichkeiten. Insgesamt gibt die Hälfte der Unternehmen an, dass sie für eine Anpassung an eine Situation mit 50 % geringerer Gasmenge weniger als ein Jahr benötigen würden. Ein Viertel rechnet jedoch mit einer Anpassungszeit von über zwei Jahren.

Die Kriegsfolgen beeinflussen überdies das Preissetzungsverhalten der Unternehmen. Die Datenlage des GBP zeigt, dass Preiserhöhungen eine direkte Antwort auf den Kostendruck sind, der insbesondere durch die Entwicklung der Energiepreise und die Versorgungsunsicherheit entsteht. Drei von vier Unternehmen in Deutschland sind hiervon finanziell betroffen. In der Folge sind Preiserhöhungen in energieintensiven Wirtschaftszweigen, insbesondere im Verarbeitenden Gewerbe und im Baugewerbe, am stärksten ausgeprägt. Dies deckt sich mit anderen Befragungsergebnissen. Kagerl et al. (2022) berichten, dass rund 70 % der Betriebsstätten von höheren Energiekosten betroffen sind, während das GBP dies für 67,4 % der Unternehmen in Deutschland beobachtet. Lieferprobleme als Grund für Kostensteigerungen werden bei Kagerl et al. (2022) für etwa 25 % der Betriebsstätten und im GBP für 32 % der Unternehmen genannt.

Insgesamt lässt sich feststellen, dass Unternehmen infolge der Pandemie und des Ukrainekriegs mit einem erhöhten Kostendruck wegen steigender Energiepreise und Lieferproblemen konfrontiert sind. Die Erhöhungen der eigenen Absatzpreise sind die erste Maßnahme, mit der Unternehmen aktuell diese höheren Kosten zu kompensieren versuchen. Weitere Preiserhöhungen an den Absatzmärkten sind insofern nur zu vermeiden, wenn es Unternehmen gelingt, auf der Kostenseite Entlastung zu schaffen. Dies hängt einerseits von globalen Entwicklungen ab, etwa dem weiteren Verlauf des Kriegs in der Ukraine oder der Unterbrechung von Lieferketten in Asien, andererseits von der Transformation der Energieversorgung in Deutschland, etwa der zuverlässigen Verfügbarkeit von alternativen Energiequellen oder der Entwicklung energiesparender Technologien.
